# Latent Membrane Protein LMP2A Impairs Recognition of EBV-Infected Cells by CD8+ T Cells

**DOI:** 10.1371/journal.ppat.1004906

**Published:** 2015-06-11

**Authors:** Chiara Rancan, Leah Schirrmann, Corinna Hüls, Reinhard Zeidler, Andreas Moosmann

**Affiliations:** 1 Clinical Cooperation Group Immunooncology, Department of Medicine III, Klinikum der Universität München, and Research Unit Gene Vectors, Helmholtz Zentrum München, Munich, Germany; 2 German Center for Infection Research (DZIF), Munich, Germany; 3 Research Unit Gene Vectors, Helmholtz Zentrum München, Munich, Germany; 4 Department of Otorhinolaryngology, Klinikum der Universität München, Munich, Germany; Northwestern University, UNITED STATES

## Abstract

The common pathogen Epstein-Barr virus (EBV) transforms normal human B cells and can cause cancer. Latent membrane protein 2A (LMP2A) of EBV supports activation and proliferation of infected B cells and is expressed in many types of EBV-associated cancer. It is not clear how latent EBV infection and cancer escape elimination by host immunity, and it is unknown whether LMP2A can influence the interaction of EBV-infected cells with the immune system. We infected primary B cells with EBV deleted for LMP2A, and established lymphoblastoid cell lines (LCLs). We found that CD8+ T cell clones showed higher reactivity against LMP2A-deficient LCLs compared to LCLs infected with complete EBV. We identified several potential mediators of this immunomodulatory effect. In the absence of LMP2A, expression of some EBV latent antigens was elevated, and cell surface expression of MHC class I was marginally increased. LMP2A-deficient LCLs produced lower amounts of IL-10, although this did not directly affect CD8+ T cell recognition. Deletion of LMP2A led to several changes in the cell surface immunophenotype of LCLs. Specifically, the agonistic NKG2D ligands MICA and ULBP4 were increased. Blocking experiments showed that NKG2D activation contributed to LCL recognition by CD8+ T cell clones. Our results demonstrate that LMP2A reduces the reactivity of CD8+ T cells against EBV-infected cells, and we identify several relevant mechanisms.

## Author Summary

Epstein-Barr virus (EBV) is carried by most humans. It can cause several types of cancer. In healthy infected people, EBV persists for life in a "latent" state in white blood cells called B cells. For infected persons to remain healthy, it is crucial that they harbor CD8-positive "killer" T cells that recognize and destroy precancerous EBV-infected cells. However, this protection is imperfect, because the virus is not eliminated from the body, and the danger of EBV-associated cancer remains. How does the virus counteract CD8+ T cell control? Here we study the effects of latent membrane protein 2A (LMP2A), which is an important viral molecule because it is present in several types of EBV-associated cancers, and in latently infected cells in healthy people. We show that LMP2A counteracts the recognition of EBV-infected B cells by antiviral killer cells. We found a number of mechanisms that are relevant to this effect. Notably, LMP2A disturbs expression of molecules on B cells that interact with NKG2D, a molecule on the surface of CD8+ T cells that aids their activation. In this way, LMP2A weakens important immune responses against EBV. Similar mechanisms may operate in different types of LMP2A-expressing cancers caused by EBV.

## Introduction

Epstein-Barr virus (EBV), which belongs to the human herpesvirus family, is a persistent virus carried by more than 90% of the adult population worldwide. EBV has a preferential B cell tropism, and latently infected B cells constitute the viral reservoir in healthy carriers [1]. Acute infection can lead to infectious mononucleosis (IM), a self-limiting lymphoproliferative disease characterized by expansion of EBV-infected B cells and virus-specific CD8+ T cells [2]. EBV is an oncovirus, and can contribute to the development of various cancers, such as Burkitt lymphoma, nasopharyngeal carcinoma and Hodgkin lymphoma [3,4]. In healthy carriers, EBV infection is under control of a diverse repertoire of antigen-specific T cells, and an important role is played by CD8+ T cells that recognize viral protein-derived peptides presented by MHC class I molecules [2]. In contrast, immunosuppressed patients who lack EBV-specific T cell responses, such as patients after transplantation, are prone to developing EBV-associated lymphoproliferative disease. This condition can be treated or prevented by transfer of EBV-specific T cells [5–7].

In immunocompetent EBV carriers, a majority of EBV-infected B cells in peripheral blood carry EBV without expressing any viral protein, a state that is called "true latency" or "latency 0" [4,8]. Thus, such latently infected B cells are invisible to EBV-specific T cells.

In contrast, during lytic EBV replication many viral proteins are expressed [9,10]. In this situation, the virus would be particularly vulnerable to immune control. Thus, EBV has evolved a number of proteins expressed in the lytic cycle that interfere with the display of viral antigens to CD8+ T cells. These proteins include BNLF2a, which inhibits the transporter of antigen processing [11], BILF1, which induces MHC class I internalization and degradation [12], and BGLF5, which inhibits cellular protein biosynthesis [13].

In proliferating infected B cells, EBV installs another program of gene expression, the "growth" or "latency III" program. This type of latency is found in *in vitro* EBV-induced lymphoblastoid cell lines (LCLs), in post-transplant lymphoproliferative diseases [14], as well as in EBV-infected B cells in lymphoid organs during primary and persistent EBV infection, where this program is thought to lead to amplification of EBV load through proliferation of infected cells [4,8]. Several immunogenic EBV antigens, the latent membrane proteins (LMP1, LMP2A, LMP2B) and the Epstein-Barr nuclear antigens (EBNA1, -2, -3A, -3B, -3C, -LP), are expressed in latency III EBV-infected B cells [9,10]. How do B cells expressing the EBV "growth program" manage to escape from recognition and elimination by virus-specific T cells? Previous studies on immunoevasion in EBV latency have focused on the nuclear protein EBNA1 or the latent membrane protein LMP1. EBNA1 interferes with its own presentation to CD8+ T cells through its glycine-alanine repeat domain [15,16], which reduces processing by the proteasome [17] and interferes *in cis* with EBNA1 translation [18–20]. As a result, presentation of EBNA1 epitopes on MHC class I to T cells is reduced. Likewise, LMP1 interferes *in cis* with presentation of its own epitopes [21]. Although several other viral proteins are expressed in the EBV growth program, it has remained unknown whether presentation to T cells of epitopes from these proteins may be suppressed by viral mechanisms.

The EBV latent protein LMP2A is a regular constituent of the EBV growth program, and is also expressed in a variety of EBV-associated cancers [9,10]. LMP2A has various functions in infected cells. Reminiscent of the accessory subunits of the B-cell receptor, the N-terminal cytoplasmic domain of LMP2A activates protein tyrosine kinases and induces downstream pathways of B cell activation [22,23]. Accordingly, LMP2A can stand in for deficient B-cell receptor signaling in mouse or human models, ensuring B cell survival [24,25]. In EBV-infected B cells, however, LMP2A counteracts lytic EBV reactivation triggered by cross-linking of the B-cell receptor [26–28]. No consensus has been reached yet on the importance of LMP2A in B cell proliferation and transformation [25,29–35].

Given these complexities, we hypothesized that LMP2A may have other functions that are not cell-intrinsic or directly related to virus replication, but related to immune control. This possibility was already suggested by the observation that LMP2A modulates signalling of type I/II interferon receptors in epithelial cells [36], that the presence of LMP2A alters the expression of several immune-related genes [37], and that LMP2A increases expression of the cytokine interleukin-10 (IL-10) [38], which may exert immunomodulatory functions. In this study, we investigated the influence of LMP2A in recognition of infected cells by immune effector cells. We show that LMP2A reduces recognition of infected B cells by EBV-specific CD8+ T cells, and we describe several mechanisms that may contribute to this effect.

## Results

We established EBV-transformed B cell lines (lymphoblastoid cell lines, LCLs) with an EBV deleted for LMP2A [25]. This virus is deleted for the promoter and the first exon of LMP2A on a background of EBV strain B95.8. Expression of LMP2B is still possible in this mutant. In line with previous findings [25,29], we found that the LMP2A-deficient virus (ΔLMP2A) had reduced efficiency of B cell transformation. To facilitate the establishment of LMP2A-deficient LCLs, we infected primary B cells with mutant EBV on a layer of murine fibroblasts overexpressing human CD40 ligand (CD40L). Infection with recombinant EBV 2089 [39] that contains the complete B95.8 EBV genome (here denoted "wild-type", WT), which is parental to the ΔLMP2A construct, was carried out in parallel under the same conditions. Outgrowing B cell cultures were expanded and maintained in the absence of CD40L stimulators. Under these conditions, WT and ΔLMP2A LCLs could be established with similar efficiency, and expanded in parallel using the same procedures. A closer analysis of established WT and ΔLMP2A LCLs showed that the rate of apoptosis was the same, but proliferation was somewhat slower in ΔLMP2A LCLs (S1 Fig). Thus, LMP2A increased the efficiency of EBV transformation *in vitro*, but was not essential for the proliferation of established LCLs.

**Fig 1:**
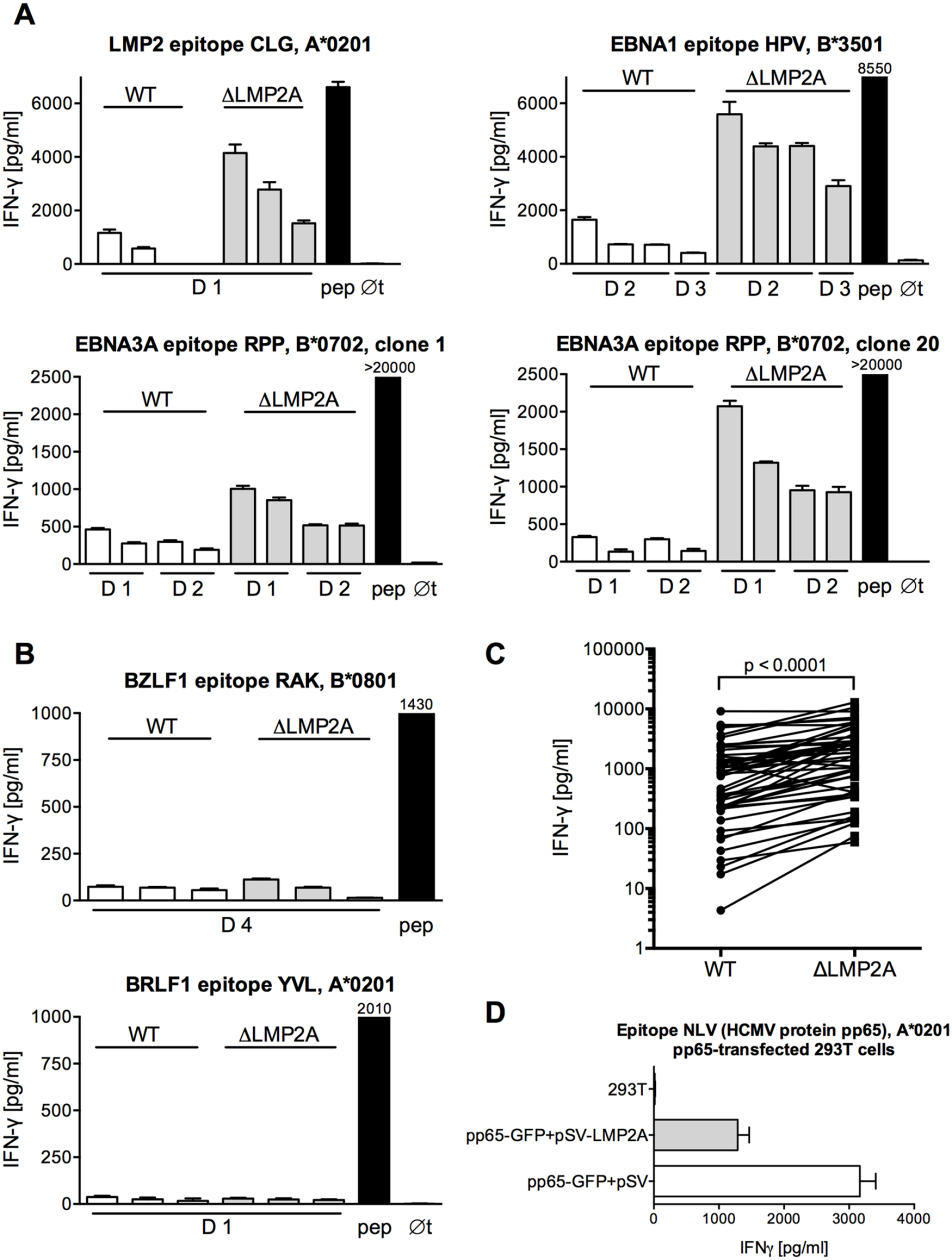
Modulation of CD8+ T cell recognition by LMP2A. (A, B) CD8+ T cell clones specific for epitopes from EBV latent antigens (A) or EBV lytic-cycle antigens (B) were co-incubated with HLA-matched LCLs that carried an EBV expressing LMP2A (WT) or deleted for LMP2A (ΔLMP2A). After 16 hours, IFN-γ in the supernatant was quantified by ELISA. Each diagram represents one experiment with a single CD8+ T cell clone and matched sets of WT and ΔLMP2A LCLs from different donors (D1, D2…). The LCLs in each donor’s set were simultaneously established and cultivated in parallel. Diagram headlines state the EBV antigen recognized by each T cell clone, the epitope peptide, and HLA class I restriction. “pep”, WT LCL exogenously loaded with the corresponding peptide; “∅t”, T cells alone. Mean + SEM for three replicates is shown. Graphs are representatives for two to five independent experiments performed for each T cell clone. (C) Synopsis of recognition of WT and ΔLMP2A LCLs by EBV-specific CD8+ T cell clones, determined by measuring IFN-γ secretion in ELISA. Each pair of values represents one EBV-specific CD8+ T cell clone tested against WT and ΔLMP2A LCLs from one donor, tested in one experiment. Where several LCLs of the same type from the same donor were tested in the same experiment, their mean is represented as one data point to allow donor-specific paired analysis (Wilcoxon signed-rank test). CD8+ T cell clones were specific for latent proteins LMP2 (peptides FLY and CLG), EBNA1 (HPV), and EBNA3A (RPP and QAK). LCLs were from 4 different donors. (D) 293T cells were co-transfected with a plasmid encoding HCMV pp65 (pp65-GFP) and a plasmid encoding LMP2A (pSV-LMP2A) or its negative control (pSV). Recognition of the pp65 epitope NLV by clonal CD8+ T cells was measured by IFN-γ ELISA after 16 hours of co-incubation. Mean + SEM is shown for two CD8+ T cell clones and duplicate transfections. One of three independent experiments is shown.

We analyzed the reactivity of EBV-specific CD8+ T cells to ΔLMP2A and WT LCLs (Fig 1). We found that CD8+ T cell clones specific for epitopes from all latent antigens tested (EBNA1, EBNA3A, LMP2) showed a higher IFN-γ release in response to ΔLMP2A LCLs than to WT LCLs (Fig 1A and 1C). CD8+ T cells specific for the LMP2 epitope CLG recognized ΔLMP2A LCLs, because the CLG peptide is derived from a transmembrane region that is shared between LMP2A and LMP2B. CD8+ T cell clones specific for lytic-cycle antigens (BRLF1, BZLF1) showed weak recognition of both types of LCLs, and therefore differences in recognition could not be detected (Fig 1B). Thus, LMP2A interferes with CD8+ T cell recognition of EBV latent antigens.

To confirm that these differences in T cell recognition were caused by LMP2A and not some other unrecognized deviations between the two EBV constructs, we tested the effect of LMP2A on CD8+ T cell recognition in isolation, in the absence of an EBV genome (Fig 1D). Co-transfection of LMP2A reduced CD8+ T cell recognition of 293T kidney cells transfected with the HCMV antigen pp65. This experiment showed that the effect of LMP2A on T cell recognition was not limited to the context of the EBV genome.

**Fig 2:**
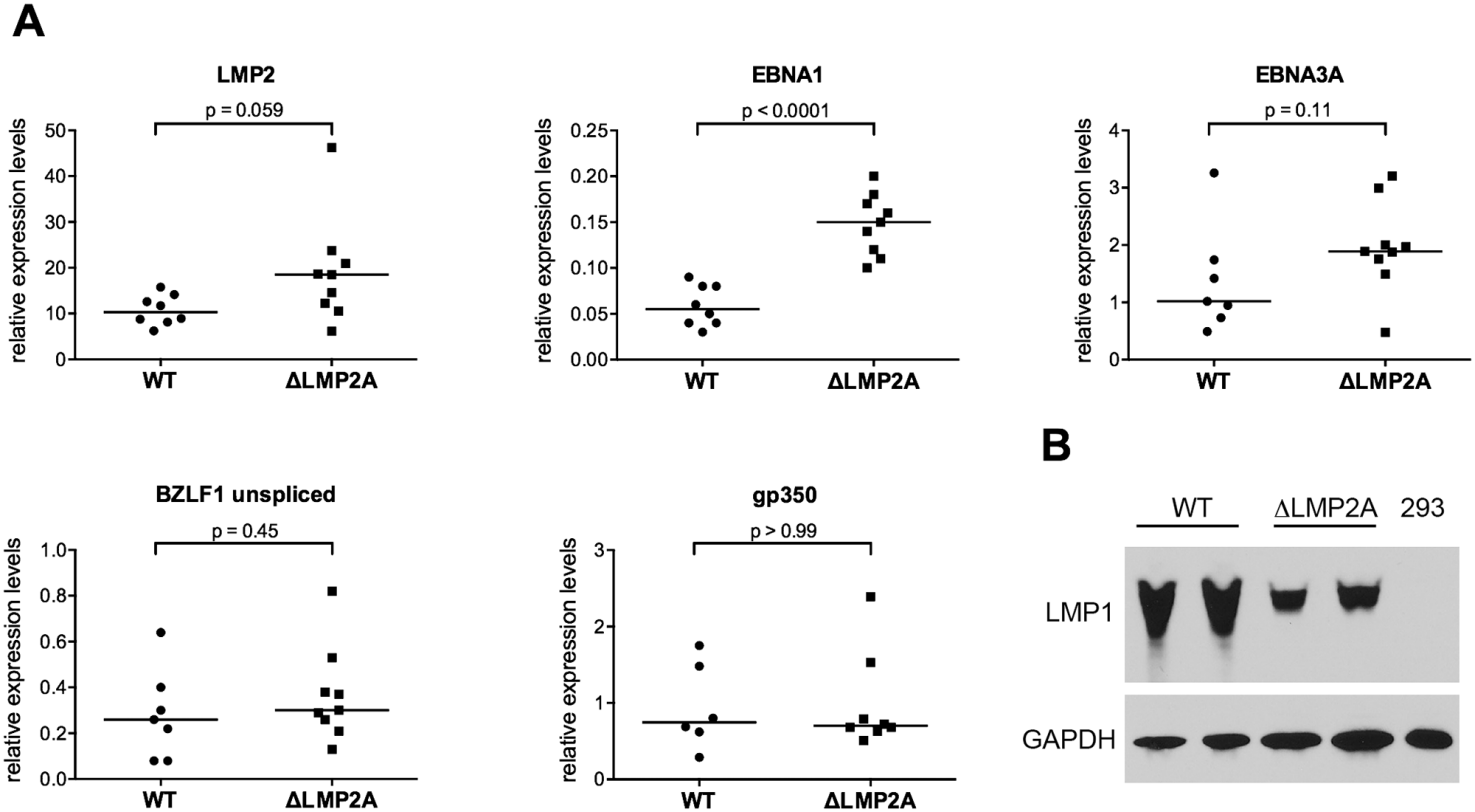
Expression of EBV latent and lytic-cycle genes in LCLs with or without LMP2A. (A) Transcript levels of LMP2, EBNA1, EBNA3A (latent genes, top), BZLF1, and gp350 (lytic-cycle genes, bottom) were measured by quantitative RT-PCR in LCLs established with WT or ΔLMP2A EBV. Expression relative to the housekeeping gene β-glucuronidase (GUSB) is shown. Within each diagram, each dot represents an independently established LCL. Per condition, six to nine independently established LCLs from 4 different donors were analyzed. From each donor, both WT and ΔLMP2A LCLs were tested. The horizontal line indicates the median. The Mann-Whitney U test was applied. (B) Expression of LMP1 was analyzed by Western blot. Two independently established WT LCLs and two independent ΔLMP2A LCLs from the same donor are shown. EBV-free 293 kidney cells serve as negative control. This is representative of experiments with 4 donors (two WT and two ΔLMP2A LCLs each).

In the early stages of infection, there are differences in EBV gene transcription in B cells carrying LMP2A-negative EBV as opposed to LMP2A-positive EBV [35]. Thus, we investigated whether the observed differences in T cell recognition of ΔLMP2A and WT LCLs were related to differential expression of EBV antigens. Average transcript levels of several EBV latent antigens (EBNA1, EBNA3A, LMP2) appeared to be increased in ΔLMP2A LCLs (Fig 2A). However, this difference reached p < 0.05 only for EBNA1. No difference between WT and ΔLMP2A LCLs was seen for median expression of the lytic-cycle genes BZLF1 and gp350. Thus, LMP2A may downmodulate the expression of some latent antigens in EBV-infected B cells, in particular EBNA1. This may contribute to the reduced presentation of these antigens to CD8+ T cells by WT LCLs.

LMP1 is an EBV protein that may alter CD8+ T cell recognition of infected cells, in particular by inducing MHC I pathway components through NF-κB, but also by inducing immunomodulatory genes [21,40]. We found that expression of LMP1 at the protein level was somewhat reduced in ΔLMP2A LCLs (Fig 2B). This argued against a possible role of LMP1 in contributing to increased recognition of ΔLMP2A LCLs by upregulating MHC I presentation.

**Fig 3:**
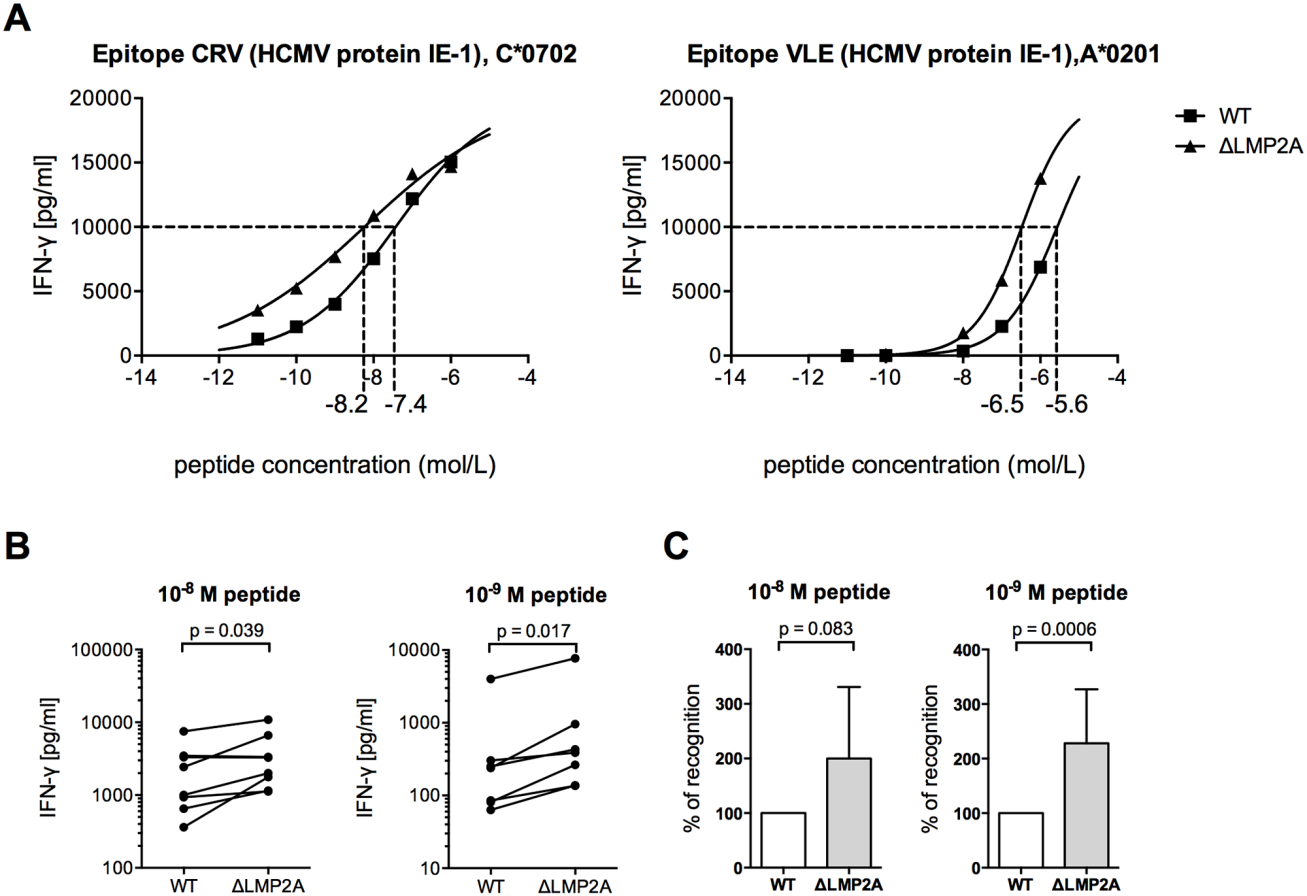
CD8+ T cell recognition of heterologous peptides on LCLs with or without LMP2A. WT and ΔLMP2A LCLs were loaded with peptides CRV and VLE from HCMV IE-1 or peptide NLV from HCMV pp65 at the indicated concentrations, and were coincubated for 16 hours with cognate antigen-specific CD8+ T cell clones. IFN-γ release by CD8+ T cells into supernatant was quantified by ELISA. (A) Exemplary dose-response curves. Dotted lines indicate the peptide concentration that induced half-maximal IFN-γ release. (B) Paired analysis of results by donor. Each pair of values represents LCLs from one donor tested in one experiment with one peptide. Three HCMV peptides and LCLs from 3 donors were included in this analysis. The Wilcoxon signed-rank test was applied. (C) Synoptic analysis of the relative change in recognition in the absence of LMP2A, setting recognition of WT LCLs to 100% for each pair of values shown in B. The Mann-Whitney U test was applied.

Next, we investigated whether LMP2A modulated the reactivity of CD8+ T cells to EBV-infected B cells by mechanisms other than altering the availability of EBV antigens. We loaded WT and ΔLMP2A LCLs exogenously with peptides CRV and VLE, derived from the human cytomegalovirus (HCMV) protein IE-1, and we analyzed LCL recognition by HCMV-specific CD8+ T cell clones (Fig 3). Peptide-loaded ΔLMP2A LCLs were more strongly recognized by these CD8+ T cells than peptide-loaded WT LCLs, resulting in higher IFN-γ release. We also investigated direct killing by cytotoxic CD8+ T cells, but did not observe differences in killing of WT and ΔLMP2A LCLs loaded with HCMV peptides (S2 Fig). The reasons for differential regulation of IFN-γ secretion and direct cytotoxicity in this setting remain to be elucidated.

Because the intracellular antigen processing machinery was bypassed in these peptide-loading experiments, LMP2A appears to act on CD8+ T cells through mechanisms other than regulation of EBV antigens or of intracellular processing pathways. Therefore, we studied the effect of LMP2A on cell surface-residing or secreted factors relevant for CD8+ T cell recognition.

**Fig 4:**
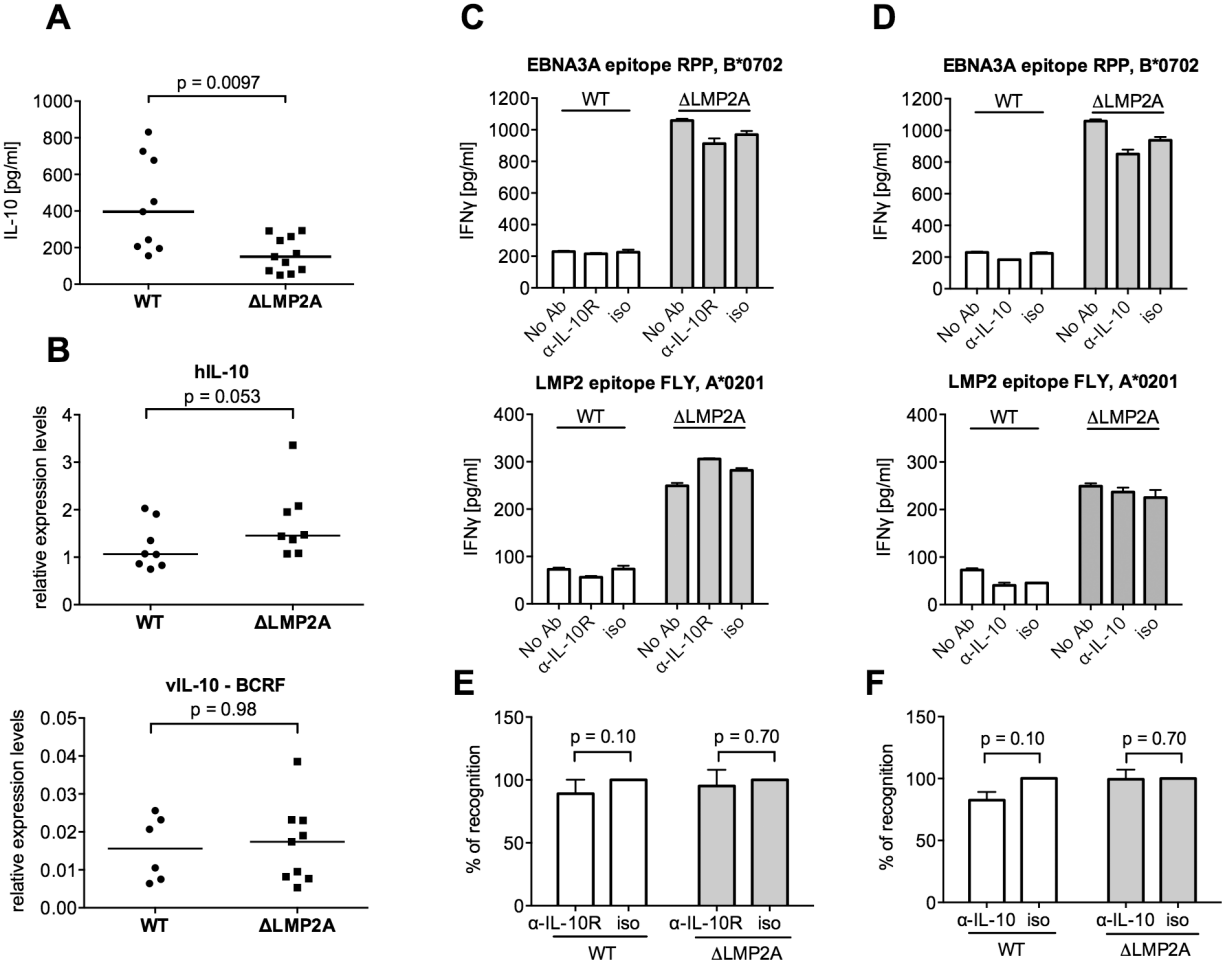
Role of LCL-secreted IL-10 in CD8+ T cell recognition of WT and ΔLMP2A LCLs. (A) WT and ΔLMP2A LCLs were washed and incubated at 0.5x10^6^ cells/ml for 18 hours, and released IL-10 was measured by ELISA. Each dot represents the mean of quadruplicates for a single independently established cell line. Data shown are representative for three independent experiments. WT and ΔLMP2A lines established from 5 different donors were analyzed in parallel. The horizontal line indicates the median; the Mann-Whitney U test was applied. (B) mRNA levels for human IL-10 and BCRF1 (viral IL-10) were measured by quantitative RT-PCR in WT and ΔLMP2A LCLs. Expression relative to the housekeeping gene GUSB is displayed. Each dot represents an independently established LCL. LCLs were from 4 different donors, WT and ΔLMP2A LCLs from each donor were analyzed in parallel. Data are representative of two independent experiments. The horizontal line indicates the median; the Mann-Whitney U test was applied. (C-F) Reactivity of EBV-specific CD8+ T cell clones against WT or ΔLMP2A LCLs was determined after antibody blocking of the IL-10 receptor on the T cells (C, E) or in the presence of a neutralizing IL-10-specific antibody (D, F). Conditions with no antibody (No Ab) or a matched isotype control antibody (iso) were tested in parallel. After 16 hours, IFN-γ release into the supernatant was quantified by ELISA. Mean and range of duplicates are shown. (C, D) Exemplary experiments. (E, F) Analyses of the relative change in recognition due to antibody blocking, with isotype controls set to 100%. A synopsis of experiments with three different T cell clones with overall mean and SEM is shown. Statistical analysis was performed with the Mann-Whitney U test.

It was recently shown that LMP2A increases IL-10 production in infected B cells [38]. The possibility of a similar effect in our system was intriguing, because cellular IL-10 and its viral homolog reduce the antiviral activity of different types of immune effector cells [41–43]. In accordance with Incrocci and colleagues [38], we found that WT LCLs released higher amounts of IL-10 than LCLs lacking LMP2A (Fig 4A). These levels of secreted IL-10 were not mirrored by transcription levels for human IL-10 (Fig 4B), which suggested an effect of LMP2A on post-transcriptional regulation of IL-10 [44]. In contrast to cellular IL-10, transcription of viral IL-10 was very low in each type of LCL (Fig 4B), in accordance with its description as a lytic-cycle gene [45]. To determine whether differences in IL-10 release could directly influence T cell reactivity to LCLs, we used specific antibodies to block IL-10 receptor on CD8+ T cells (Fig 4C and 4E), or to neutralize IL-10 in the supernatant (Fig 4D and 4F). In each case, recognition of WT or ΔLMP2A LCLs was not altered. Thus, modulation of IL-10 secretion by LMP2A did not directly affect the ability of CD8+ T cells to recognize infected B cells. This experiment did not rule out indirect effects of secreted IL-10, which may act back on the LCLs over time in culture and modulate their immunogenicity.

**Fig 5:**
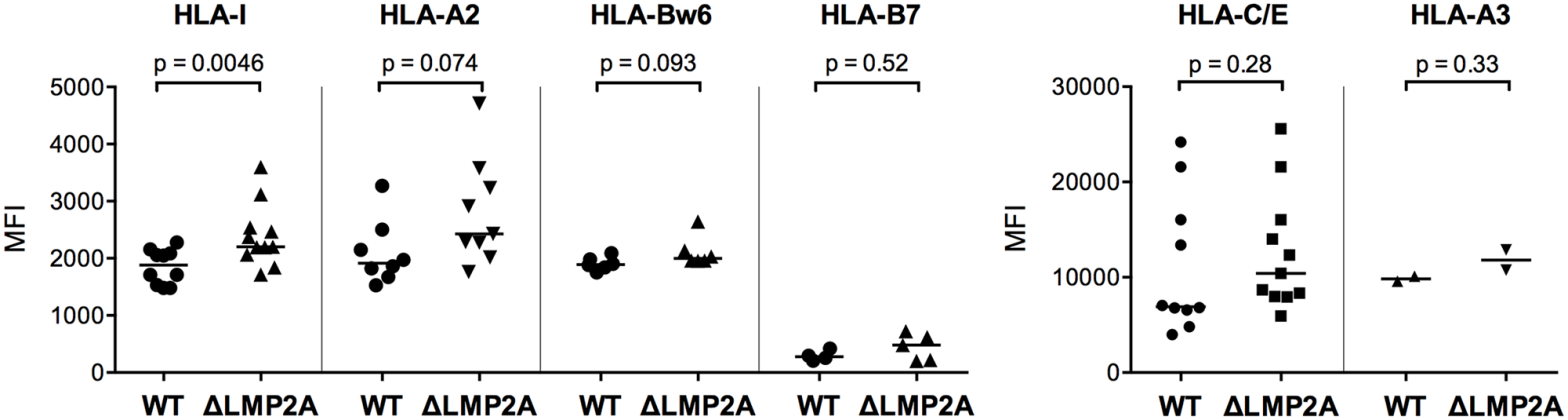
Expression of MHC class I on the surface of WT and ΔLMP2A LCLs. LCLs were stained with antibodies specific for total HLA class I (“HLA-I”) or HLA class I allotypes as indicated, and analyzed by flow cytometry. Each dot represents a single measurement for a single independently established cell line from one of 5 different donors. Data are representative for three independent experiments. MFI, mean fluorescent intensity. The horizontal line indicates the median. The Mann-Whitney U test was used.

We continued by analyzing ΔLMP2A and WT LCLs for cell surface molecules involved in the interaction between CD8+ T cells and LCLs. First, we determined the levels of total MHC-I and individual MHC-I allotypes (Fig 5). MHC-I was marginally increased in LCLs deleted for LMP2A as compared with WT LCLs (p = 0.0046). A similar tendency was observed for some of the individual MHC-I allotypes, but did not reach p < 0.05.

**Fig 6:**
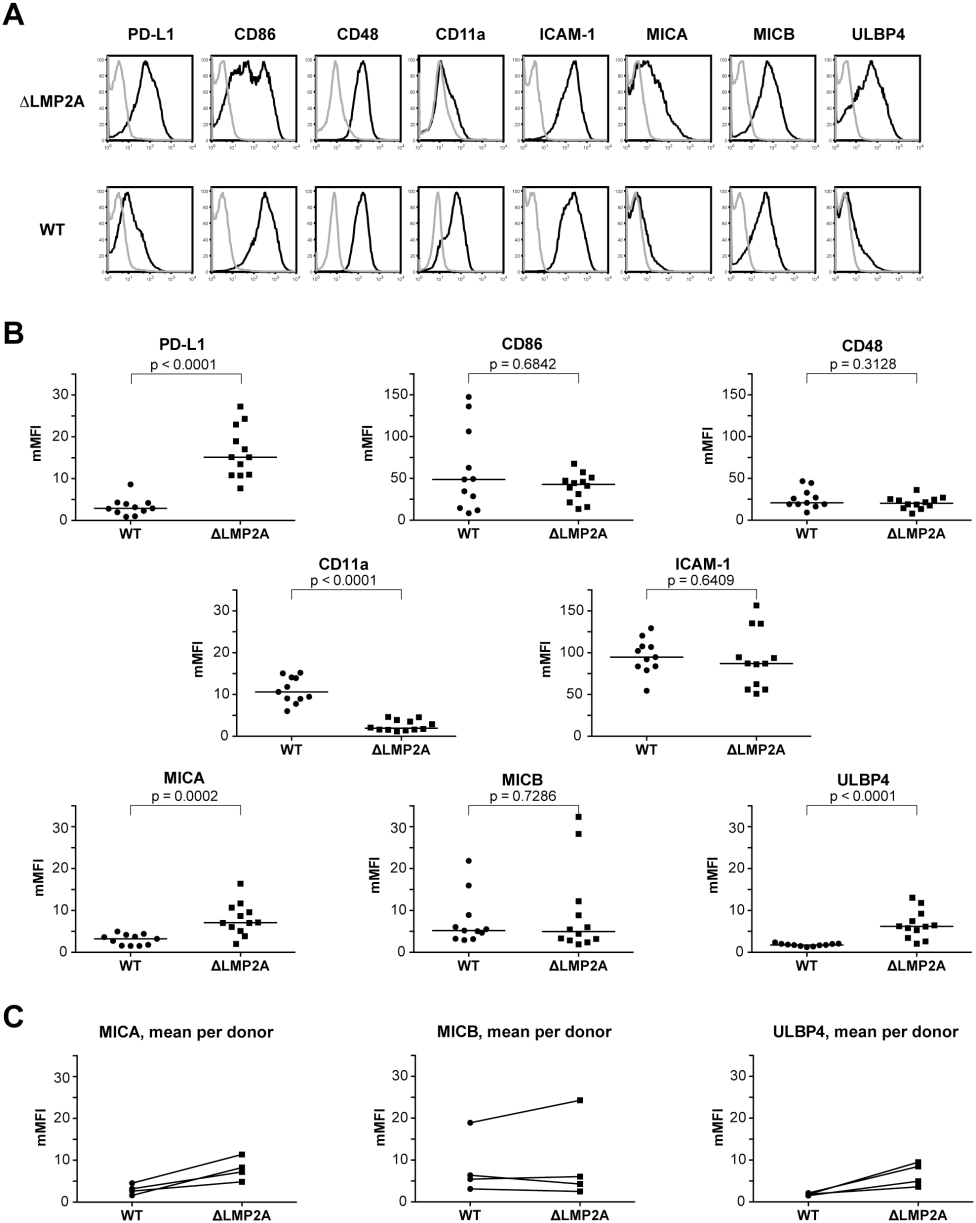
Immunophenotype of EBV-infected B cells with or without LMP2A. **(A, B)** Expression of surface molecules on WT and ΔLMP2A LCLs was determined by flow cytometry after staining with monoclonal antibodies. (A) Representative histograms for one WT LCL and one ΔLMP2A LCL from the same donor. Black line, specific antibody; grey line, isotype control. (B) Multiplicity of mean fluorescence intensity (mMFI) was calculated by dividing MFI of specific antibody by MFI of isotype control. Each dot represents a single analysis of an independently generated LCL. WT and ΔLMP2A LCLs from the same donors were examined in parallel, and LCLs from 4 donors were concurrently analyzed. The horizontal line indicates the median, the Mann-Whitney U test was applied. (C) Paired analysis of NKG2D ligand expression by donor.

Next, we examined whether expression of selected costimulatory and immunomodulatory molecules on the surface of LCLs was altered in the absence of LMP2A (Fig 6). We found strong differences in expression for some of these molecules. The coinhibitory B7 family molecule PD-L1 (B7-H1) was (somewhat unexpectedly) induced in ΔLMP2A LCLs, whereas the costimulatory B7 molecule CD86 was equally expressed on ΔLMP2A and WT LCLs. CD11a, the α chain of the integrin LFA-1 that plays important roles in the immunological synapse, was strongly downregulated in the absence of LMP2A, whereas ICAM-1 (CD54), its counterpart, was expressed equally in the presence or absence of LMP2A. So far, these alterations were not obviously connected with the increased susceptibility of ΔLMP2A cells to CD8+ T cell recognition.

Recent reports suggested that EBV infection induces ligands of the coactivatory receptor NKG2D, a molecule expressed on T and NK cells [46–49]. However, a comprehensive analysis of NKG2D ligands on LCLs has not previously been performed. Our analyses by flow cytometry showed that EBV infection induced the expression of three NKG2D ligands (MICA, MICB and ULBP4) on LCLs (Fig 6). These molecules were not expressed on primary B cells. Markedly higher levels of MICA and ULBP4 were detected on ΔLMP2A LCLs as compared to WT LCLs, whereas MICB levels did not differ (Fig 6). We could not detect expression of the other five NKG2D ligands (ULBP1, 2, 3, 5, 6) on the surface of WT or ΔLMP2A LCLs with available monoclonal antibodies, but this does not rule out that these molecules may as well be modulated by LMP2A. Our results suggested a possible contribution of NKG2D ligands to differential recognition of LCLs by CD8+ T cells.

**Fig 7:**
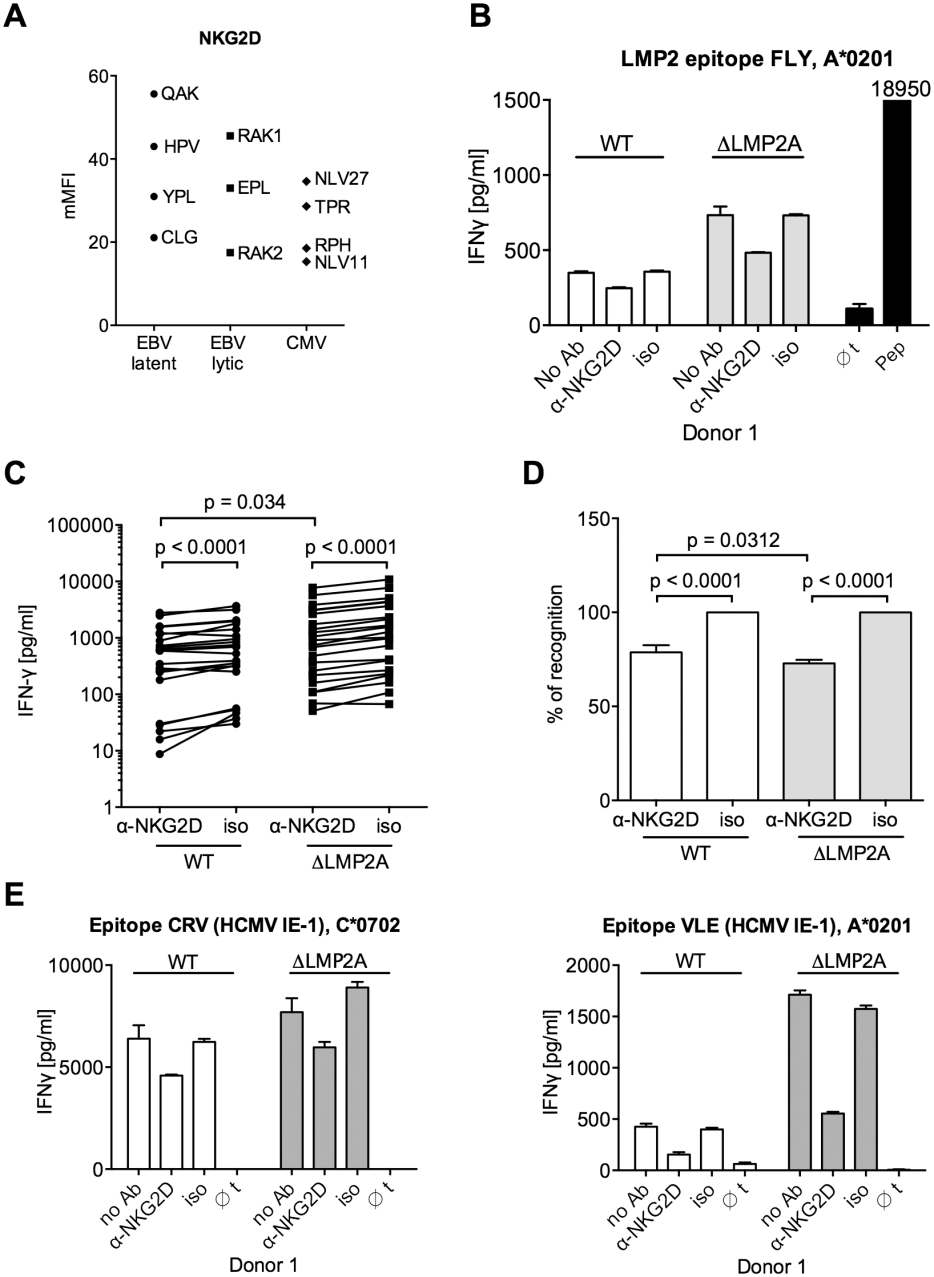
Effect of NKG2D blocking on CD8+ T cell recognition of LCLs with or without LMP2A. (A) Expression of NKG2D on CD8+ T cell clones specific for a variety of EBV or HCMV epitopes. Specificity is indicated by the first three amino acids of the peptide. Each dot represents a single analysis of one CD8+ T cell clone. (B–E) CD8+ T cell clones were co-incubated with WT or ΔLMP2A LCLs in the presence of an anti-NKG2D antibody (α-NKG2D), a matched isotype control (iso), or no antibody (No Ab). T cells had been pre-treated with the antibodies for 1 hour before incubation with LCLs. After 16 hours, IFN-γ in the supernatant was evaluated by ELISA. (B) A representative experiment for a CD8+ T cell clone specific for the FLY epitope from EBV latent antigen LMP2. “pep”, WT LCL exogenously loaded with corresponding peptide as a positive control; “∅t”, T cells alone. Mean and range of duplicates are shown. (C) Summary of experiments with EBV-specific CD8+ T cell clones. Epitopes from the latent proteins LMP2 (FLY and CLG), EBNA1 (HPV) and EBNA3A (RPP) were tested. Each pair of values linked by a line represents the same LCL, treated with α-NKG2D or isotype control antibody, and here the Wilcoxon signed-rank test was applied. A total of six LCL donors were analyzed. WT and ΔLMP2A LCLs from the same donor were always analyzed in parallel. For comparison of WT and ΔLMP2A LCLs, the Mann-Whitney U test was used. (D) Relative change of recognition upon NKG2D blocking. Isotype controls were set to 100% recognition. Statistical analysis was performed as in C. (E) WT and ΔLMP2A LCLs were loaded with peptides CRV and VLE from the HCMV protein IE-1 at 10^–8^ M. Antibody blocking and analysis of specific T cell recognition were performed as above.

We tested the functional relevance of differential NKG2D ligand expression for CD8+ T cell recognition. An analysis of NKG2D levels on several CD8+ T cell clones showed that all were positive for NKG2D (Fig 7A). Differences in NKG2D expression levels were not correlated with antigen specificity. When we blocked NKG2D on EBV-specific CD8+ T cells with a specific antibody, IFN-γ release after contact with LCLs was reduced (Fig 7B–7D). A reduction in the reactivity of CD8+ T cells to both WT and mutant LCLs was observed after blocking, but reduction was even slightly stronger for ΔLMP2A LCLs than for WT LCLs (Fig 7C and 7D). Likewise, blocking NKG2D on HCMV-specific CD8+ T cell clones led to reduced recognition of peptide-loaded LCLs (Fig 7E). Thus, NKG2D ligands on LCLs contribute to their recognition by CD8+ T cells irrespective of antigen specificity. LMP2A reduces CD8+ T cell recognition of EBV-infected B cells by reducing the expression of NKG2D ligands.

**Fig 8:**
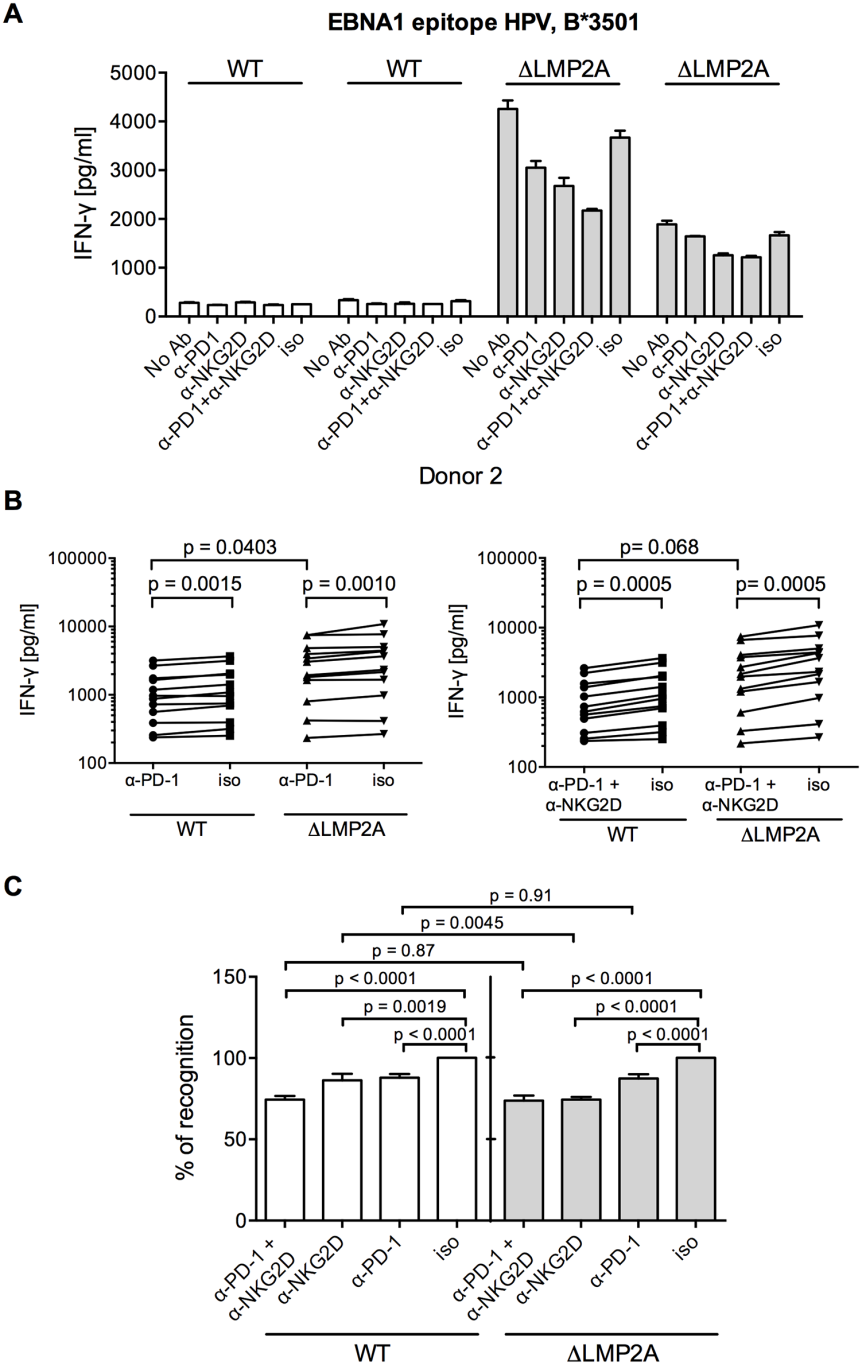
Effect of PD-1 blocking on CD8+ T cell recognition of LCLs with or without LMP2A. (A) A CD8+ T cell clone specific for the HPV epitope from EBV latent antigen EBNA1 was co-incubated with WT and ΔLMP2A LCLs from donor 2 in the presence of a blocking α-PD-1 antibody, a blocking α-NKG2D antibody, a combination of the two, a matched isotype control (iso), or no antibody (No Ab). T cells were pre-treated with the antibodies for 1 hour before and during 16 hours of coincubation with LCLs. Afterwards, IFN-γ in the supernatant was evaluated by ELISA. Mean and range of duplicates are shown. (B) Summary of experiments with PD-1 blockade or combined PD-1/NKG2D blockade. EBV-specific CD8+ T cell clones were specific for peptides FLY and CLG from LMP2 (FLY and CLG) and HPV from EBNA1, LCLs from 4 donors were used as targets. Each dot represents the mean of three experimental replicates of one T cell clone incubated with one LCL. WT and ΔLMP2A LCLs from the same donor were always tested in parallel. Specific antibody versus isotype control was evaluated with the Wilcoxon signed-rank test, WT versus ΔLMP2A LCLs with the Mann-Whitney U test. (C) Relative change in recognition upon PD-1, NKG2D or combined blocking. IFN-γ release in the presence of isotype control (iso) was set to 100%. Specific antibody versus isotype was evaluated with the Wilcoxon test, WT versus ΔLMP2A LCLs with the Mann-Whitney U test.

Since expression of PD-L1, a ligand of the immunomodulatory receptor PD-1 on T cells, was increased on ΔLMP2A LCLs (Fig 6), the question emerged whether PD-L1 may counteract T cell recognition of ΔLMP2A LCLs. In this case, even greater differences in T cell recognition of ΔLMP2A LCLs as opposed to WT LCLs might be revealed by masking the effects of PD-L1. Since it was reported that stimulation of PD-L1 on LCLs induces their apoptosis in a T-cell-independent manner [50], we used a PD-1-blocking antibody, EH12.2H7, that was described to interfere with T-cell-inhibitory interactions of PD-L1 and PD-1 [51]. Thus, we tested blocking antibodies to NKG2D and PD-1 in T cell recognition assays. Interestingly, blockade of PD-1 did not increase T cell recognition of ΔLMP2A LCLs, but reduced it, although less so than blockade of NKG2D (Fig 8). Addition of PD-1 antibody to NKG2D antibody did not further modify recognition of ΔLMP2A LCLs, although recognition of WT LCLs was additively reduced by the two antibodies. We conclude that the increased amounts of PD-L1 on ΔLMP2A LCLs did not counteract T cell recognition and resulting IFN-γ production.

## Discussion

In this report, we show that LMP2A interferes with CD8+ T cell recognition of latently infected B cells, and identify several mechanisms that may contribute to this interference. First, we found that LMP2A decreased mRNA expression levels of EBV latent antigens targeted by CD8+ T cells, in particular EBNA1. Second, LMP2A downregulated MHC class I, although to a limited extent. Third, two ligands of the coactivatory receptor NKG2D were strongly upregulated in LMP2A-deficient LCLs, and blocking of NKG2D reduced T cell recognition of infected cells. We conclude that LMP2A hampers CD8+ T cell recognition of infected cells through different mechanisms including regulation of NKG2D ligands.

A basis for the present work was the efficient generation of ΔLMP2A LCLs. The importance of LMP2A for human B cell transformation by EBV has been controversial: some studies did not identify a role of LMP2A [30–34], but others reported that LMP2A increases B cell proliferation and transformation [25,29,35]. In our experience, LMP2A is important for establishment of EBV latent infection *in vitro*, as we found it difficult to establish ΔLMP2A LCLs under standard conditions. However, when we supplemented a strong CD40 stimulus for the first days after infection [52,53], ΔLMP2A LCLs and WT LCLs could be generated with similar yield, and ΔLMP2A LCLs could then be maintained autonomously. This finding confirmed that LMP2A is not essential for maintenance and proliferation of established LCLs, as long as the B cell receptor is expressed [25].

Another important function of LMP2A is its role in "stabilizing latency", i.e. prevention of lytic-cycle induction. A complex picture has emerged, in which LMP2A interferes with lytic induction after exogenous B-cell receptor stimulation [26–28,30,54], but induces basal levels of lytic gene expression in the absence of such a stimulus [55]. In accordance with earlier results [35], we found that baseline lytic gene expression was low both in WT and ΔLMP2A, and therefore was without consequence for recognition by T cells [2,56]. Thus, the "latency-stabilizing" function of LMP2A does not appear to be relevant for T cell recognition of B cells that express the growth program in the absence of exogenous triggers. It remains to be investigated whether the immunomodulatory functions of LMP2A extend to cells in lytic cycle.

Two EBV latent proteins, EBNA1 and LMP1, were previously shown to modulate antigen presentation to CD8+ T cells. EBNA1 does not affect presentation of other antigens, but specifically interferes *in cis* with its own presentation by blocking its own proteasomal processing [17] and modulating its translation [19,20], both through its glycine-alanine-rich domain. LMP1 is a strong inducer of MHC I presentation through activation of the NF-κB pathway [21,57], but contains a structural element that acts *in cis* to protect epitopes derived from its own sequence from efficient presentation [21]. LMP1 is also unusual in that peptides derived from secreted LMP1 were shown to interfere with T cell activation [58]. However, this requires amounts of LMP1 that are much higher than those secreted by EBV-infected cells [58]. To our knowledge, it has remained untested whether EBV latent antigens more generally affect recognition by and activation of CD8+ T cells.

Suggestions regarding a role of LMP2A in T immune modulation emerged from comprehensive microarray-based analyses of LMP2A-mediated changes to the transcriptome of mouse and human B cells [37]. Interestingly, transcription of genes in the inflammation/immunity category, including interferon-regulating factors, was repressed by LMP2A in human LCLs, whereas no such genes were induced [37]. Among genes of direct relevance for the B-cell—T-cell interface, CD86 was induced and LFA-1 was repressed by LMP2A in BJAB cells, but no differential expression of these genes was found in LCLs with or without LMP2A [37]. These findings highlight that the effects of LMP2A depend on the cellular context, and that T-cell-modulatory functions of LMP2A in more restricted modes of EBV latency may hypothetically be even stronger than in the LCL system studied here. For example, the ability of LMP2A to interfere with signaling through interferon receptors [36] may further contribute to LMP2A-mediated evasion from T cell recognition.

Our data demonstrated that LMP2A markedly reduced the reactivity of EBV-specific CD8+ T cells against LCLs. This was true for all latent EBV antigens investigated (LMP2, EBNA1, EBNA3A). The epitopes we analyzed are processed by different pathways for their presentation: CLG and FLY are TAP-independent epitopes, with FLY being immunoproteasome-dependent [59,60], while RPP and HPV are TAP-dependent [61]. A reduction of CD8+ T cell reactivity was also observed on LCLs loaded with exogenous peptides from a different pathogen, which makes it clear that LMP2A does not exclusively affect intracellular mechanisms of antigen provision and presentation. Reduced CD8+ T cell reactivity in the presence of LMP2A was observed in the context of all HLA allotypes that were studied: HLA A*0201, B*0702, and B*3501 for intracellularly processed EBV epitopes, HLA A*0201 and C*0702 for exogenously loaded HCMV epitopes. Thus, our data indicate that LMP2A affects CD8+ T cell reactivity regardless of the identity of the peptide presented, the mechanism of processing, or the presenting HLA molecule.

However, our results also suggested an antigen-specific aspect to the immunomodulatory effects of LMP2A, because we found a trend toward elevated expression of several latent genes in the absence of LMP2A. This is in line with the idea that LMP2A may mediate global B-cell transcription factor regulation to reduce expression of EBV latency proteins [62,63]. This was not true for LMP1, however, whose protein expression in the absence of LMP2A was reduced. Our findings are in line with similar tendencies in EBV latent gene expression in the first seven days after B cell infection with EBV ΔLMP2A [35]. It is intriguing that EBNA1 was the EBV latent antigen whose mRNA expression was most clearly reduced by LMP2A, since both antigens are part of the restricted EBV gene expression spectrum in latency II EBV malignancies such as nasopharyngeal carcinoma and Hodgkin lymphoma [64,65]. If LMP2A interferes with presentation of EBNA1-derived and other peptides also in latency II type cancers, this will have important implications for their immune surveillance.

Among immunomodulatory cytokines, IL-10 was a particularly interesting candidate in our context, because it is constitutively produced at high levels by EBV-transformed B cells [66,67], and a recent report showed that LMP2A increased IL-10 production in Burkitt lymphoma cell lines [38]. Moreover, EBV encodes a viral homologue of human IL-10 [68]. Both human and viral IL-10 were suggested early on to interfere with cellular immune responses to EBV [41,69], but it may be difficult to distinguish an immunomodulatory function of cellular or viral IL-10 from their function as growth factors for EBV-infected B cells [66,70,71]. vIL-10 contributes to downregulation of the transporter of antigen processing 1 (TAP1) and MHC-I in the early phase of B cell infection [43], but recognition of early-infected B cells by EBV-specific CD8+ T cells was not increased in the absence of vIL-10 [42]. Our data showed that LCLs lacking LMP2A released lower amounts of IL-10 compared to WT LCLs, but reactivity of CD8+ T cell clones was not altered by neutralization of IL-10 or blocking of the IL-10 receptor. However, a more indirect role of IL-10 remains possible. Therefore, LCL-secreted IL-10 may act back on the LCLs over time, and thus downregulate MHC-I or other relevant molecules [43,72] in WT LCLs more strongly than in ΔLMP2A LCLs.

PD-1 is an immunomodulatory receptor found on activated T cells, on exhausted virus-specific T cells in chronic infection, but also on functional EBV-specific effector memory T cells in latent infection [73]. Blocking of the interaction between PD-1 and its ligand, PD-L1, may restore antiviral T cell function [74]. Somewhat counter-intuitively, we found PD-L1 to be downregulated in LCLs in the presence of LMP2A. When we blocked PD-1 on CD8+ T cells, we did not observe increased reactivity to LCLs, but rather a reduction in reactivity. The possibility remains that PD-L1 on EBV-infected B cells exerts a more long-term influence on shaping specific CD8+ T cell repertoires and functions that was not tested in our experiments.

NKG2D is an agonistic receptor on T and NK cells and recognizes a number of ligands that are upregulated on target cells in conditions such as malignant transformation, viral infection or heat shock [75]. Increased expression of some NKG2D ligands after EBV infection was described [46–48,76], but a comprehensive analysis of NKG2D ligands on LCLs has been lacking. Our analysis of the eight known NKG2D ligands showed that EBV infection induced the expression of three of them (MICA, MICB, and ULBP4), and that induction of MICA and ULBP4 was even more increased in the absence of LMP2A. In addition, we demonstrated that blocking of NKG2D on CD8+ T cells distinctly affected the recognition of LCLs by these effector cells. A recent study has shown that in patients with genetic deficiencies in the magnesium transporter MAGT1, who are particularly susceptible to EBV infection and EBV+ lymphomas, NKG2D plays an important role in the control of EBV infection by NK and CD8+ T cells [46]. A role for NKG2D in control of EBV-associated cancer has been further illustrated in a mouse model of LMP1-induced cancer that could be therapeutically targeted through NKG2D [76]. Targeting of the NKG2D ligand MICB by an EBV-encoded miRNA may decrease susceptibility of EBV-infected B cells to lysis by NK cells [77]. Thus, NKG2D ligands represent important coagonists for EBV-specific adaptive and innate immunity, and it appears an efficient strategy for the virus to blunt cellular immune responses by decreasing surface expression of NKG2D ligands through the action of LMP2A.

Taken together, we describe here a functional immunomodulatory effect for the EBV protein LMP2A, and show that LMP2A mediates partial escape of infected B cells from recognition by CD8+ T cells. Several immunoevasive mechanisms are driven by LMP2A in EBV-infected B cells. Thus, it will be urgent to determine the role played by LMP2A in evasion from T and NK cell recognition in other modes of EBV infection, and in different types of EBV-associated lymphoproliferative and malignant diseases.

## Materials and Methods

### Ethics statement

Mononuclear cells were isolated from standard blood donations by anonymous healthy adult donors purchased in the form of buffy coats from the Institute for Transfusion Medicine, University of Ulm, Germany, or from voluntary healthy adult blood donors providing written informed consent. The institutional review board (Ethikkommission, Klinikum der Universität München, Grosshadern, Munich, Germany) approved this procedure. All work was conducted according to the principles expressed in the Helsinki Declaration.

### Virus production

Virus-producing cell lines for recombinant EBV 2089 (EBV WT), derived from EBV strain B95.8 [39], and its ΔLMP2A-deleted derivative EBV 2525 [25] were provided by Wolfgang Hammerschmidt [25,39]. In 293HEK-derived EBV producer cell lines, which stably carry the EBV genome in an episomal form, the viral lytic cycle was induced by transient transfection of expression plasmids coding for transcription factor BZLF1 and glycoprotein gp110/BALF4 [78]. EBV-containing supernatant was harvested three days later, centrifuged to reduce cellular debris, filtered (0.8 μM), and stored at 4°C. Titer of infectious virus was determined by infecting Raji cells for three days and quantifying GFP-positive cells as described [78]. Infection of B cells was performed at 0.1 virus units per cell.

### B cells and LCLs

Standard cell culture medium was RPMI 1640 with 10% foetal bovine serum, 100 U/ml penicillin, 100 μg/ml streptomycin (all from Invitrogen), and 100nM sodium selenite (ICN). Stimulator cell line LL8 was generated by stable transfection of L929 mouse fibroblasts with an expression plasmid for human CD40 ligand carrying a G418-selectable marker, followed by two rounds of single-cell cloning under selection. We found this stimulator cell line to be functionally analogous to the one described earlier [53,79]. Lymphoblastoid cell lines were established from primary B cells purified from freshly isolated PBMCs. Untouched B cells were negatively isolated using Human B Cell Isolation Kit II (Miltenyi Biotec, Bergisch Gladbach, Germany). Enrichment of B cells was verified by flow cytometry (anti-CD19 clone HIB19; anti-CD3 clone HIT3a, Biolegend), and was in the range of 95–98%. B cells were plated at 1×10^5^ cells/well in 24-well plates on an adherent cell layer of irradiated (180 Gy) CD40 ligand-expressing LL8 cells in standard medium supplemented with 1 mg/mL cyclosporine A (Novartis). B cells were infected with 0.1 virus units per cell. Half of the culture supernatant was exchanged at day 1 post infection. Outgrowing cultures were transferred after 1–2 weeks to a fresh plate, and further cultivated autonomously in the absence of LL8 cells. Presence of mutant EBV and absence of endogenous EBV wild type was confirmed by PCR every few weeks with primers L2BRC (5'-GCTTCCTCGTGCTTTACGGTATC-3') and L2BRD (5'-AAGAACTTTGACCTGTTGTCCCTG-3') for amplification of a product bridging the LMP2A deletion, primers L2INA (5'-CATTGCGGGTGGATAGCCTC-3') and L2BRD for amplification of the deleted sequence. Proliferating EBV-infected LCLs were analyzed and used in T cell assays between 1.5 and 4 months after infection.

### DNA transfection

DNA transfection experiments were performed with 293T human embryonic kidney cells with a plasmid expressing HCMV pp65 fused to a destabilized green fluorescent protein (GFP) under the HCMV immediate-early promoter (provided by Manuel Albanese and Wolfgang Hammerschmidt) (pp65-GFP). A plasmid expressing full-length LMP2A under the control of the SV40 early promoter (pSV-LMP2A) and the corresponding control (pSV) were used for co-transfection. The pp65-GFP plasmid and pSV plasmids were used at a 1:10 ratio. One day before transfection, 293T cells were plated in 24-well plates (1×10^5^ cells/well). Transfection was performed with a mix of 1.17 μl of Metafectene Pro (Biontex) and 390 ng of plasmid DNA in a volume of 100 μl of OptiMEM (Gibco) for each well, according to the manufacturer's protocol. Two days after transfection, cells were used for T cell assays and assessment of transfection efficiency by FACS. About 40–50% of cells were GFP-positive.

### T cell clones

To identify EBV-positive donors among anonymously purchased buffy coats, serum-containing cell-free supernatant was tested for IgG specific for EBV EBNA1 and VCA by a rapid immunofiltration assay (RDT EBV IgG Assay, Bio-Rad). EBV-specific T cells were directly isolated from PBMCs of EBV-seropositive, HLA-typed donors after stimulation with a matched peptide and IFN-γ secretion assay (Miltenyi Biotec). For single T cell cloning, isolated IFN-γ-secreting cells were seeded into round-bottom 96-well plates at 0.7 or 2.5 cells per well in 200 μl of medium supplemented with 1000 U/mL rIL-2, 1×10^5^/mL irradiated (50 Gy) HLA-matched LCLs, and 1.5×10^6^/mL of a mixture of irradiated (50 Gy) allogeneic PBMCs from at least three different donors. Outgrowing T cell clones were expanded in round-bottom 96-well plates by restimulating every 2 weeks under the same conditions. The specificity of the T cell clones was determined by IFN-γ ELISA with individual antigenic peptides (see below), and by staining with HLA-peptide pentamers (Proimmune, Oxford, UK). The T cell clones were specific for the following epitope peptides, abbreviated by their first three amino acids in one-letter code: CLG (CLGGLLTMV, LMP2, A*0201) [80], YVL (YVLDHLIVV, BRLF1 A*0201) [81], FLY (FLYALALLL, LMP2, A*0201) [60,82], RPP (RPPIFIRRL, EBNA3A, B*0702) [83], RAK (RAKFKQLL, BZLF1, B*0801) [84], HPV (HPVGEADYFEY, EBNA1, B*3501) [85]. HCMV-specific CD8+ T cells clones specific for NLV (NLVPMVATV, pp65, A*0201) [86], CRV (CRVLCCYVL, IE-1, C*0702) [87] and VLE (VLEETSVML, IE-1, A*0201) [88] were obtained as described [87].

### Flow cytometry

Flow cytometric analysis was performed with a BD FACS Calibur or a BD LSR Fortessa machine. Analysis of WT and mutant LCLs lacking LMP2A established from the same donor was always conducted in parallel and for at least one WT line and one ΔLMP2A line. Generally, 1–1.5×10^5^ cells were stained in a V-bottom 96-well plate at 4°C for 20 minutes in a volume of 20 μl, washed twice in 200 μl of buffer (PBS + 2% FCS), resuspended in buffer, and analyzed immediately. When unlabeled antibodies were included in the staining that required counterstaining with labeled anti-Ig antibodies, two to three rounds of staining and washing were performed as necessary. Antibodies anti-MICA (clone 159227, unlabeled), anti-MICB (clone 236511, unlabeled), and anti-ULBP1 (clone 170818, unlabeled) were from R&D Systems and were counterstained with anti-IgG-APC (clone Poly4053) from Biolegend. Anti-ULBP2/-5/-6 (clone 165903, PE-labeled) and anti-ULBP3 (clone 166510, PE-labeled), were from R&D Systems; anti-ULBP4 (6E6, unlabeled) was from Santa Cruz; anti-CD11a (clone G43-25B, PE-labeled) was from BD Bioscience; anti-CD48 (clone 5F4, PE-labeled), anti-PD-L1 (clone 29E.2A3, APC-labeled), anti-CD86 (clone IT2.2, APC-labeled), anti-ICAM-1 (clone HCD54, APC-labeled), anti-HLA-ABC (clone W6/32, APC-labeled), and anti-HLA-A2 (clone BB7.2, PE-labeled) were from Biolegend; and anti-HLA-B7 (clone BB7.1) was from Millipore. Antibody anti-HLA-A3 (clone 4i87, IgM, USB) was counterstained with anti-IgM-PE (clone RMM-1; BioLegend). A hybridoma producing the HLA-C/HLA-E-specific antibody DT9 (IgG2b) was kindly provided by Véronique Braud, Nice, France [89], and counterstained with anti-mouse IgG-APC (clone Poly4053) from Biolegend. HLA-Bw6 was stained with a PE-labeled human antibody (REA143, 130-099-843) from Miltenyi Biotec. Isotype controls were IgG2A (clone 133304) and IgG2B (clone 133303) from R&D Systems; IgG1 (clone MOPC-21), IgG2A (clone MOPC-173), and IgG2B (clone MG2b-57) from Biolegend.

T cells were stained with antibodies anti-NKG2D (1D11, APC-labelled), anti-CD8 (RPA-T8, Pacific Blue- or FITC-labelled), anti-CD3 (HIT3a, PE-Cy5-labelled) from BioLegend.

Combined analysis of proliferation and apoptosis of LCLs was performed using CellTrace Violet (Life Technologies), AnnexinV-Cy5 conjugate (ApoScreen, Southern Biotech), and propidium iodide (PI, Life Technologies). One million cells was stained with 1 μl of CellTrace Violet in 1 ml PBS, washed, cultivated in 3 ml of full medium in a 12 well plate at 1x10^6^ cells/well, and incubated for 4 days. Cells were harvested, counted, and 2.5×10^5^ cells were stained in 200 μl buffer with 2 μl of AnnexinV-Cy5 and propidium iodide at 1 μg/ml, before proceeding to flow cytometric analysis.

### T cell effector assays

For IFN-γ ELISA, effector cells (2.5×10^4^ cells/well) and target cells (5×10^4^ cells/well) were co-cultivated in 200 μl/well in a V-bottom 96-well plate at 37°C and 5% CO_2_. For IL-10 ELISA, LCLs were plated at 5×10^5^ cells/ml in a 12-well or V-bottom 96-well plate and incubated at 37°C and 5% CO_2_. Supernatants were harvested after 16–18 hours. ELISA was performed according to the manufacturer's instructions (Mabtech, Nacka, Sweden). Blocking by specific purified antibodies was performed where indicated. Antibody was added to the effector cells (anti-NKG2D, anti-IL10R) or to the target cells (anti-IL10) at a pre-established concentration and incubated for 1 hour at 37°C prior to the addition of the target or effector cells, respectively. We used antibodies to IL-10 and IL-10R that were previously shown to neutralize activity at the same or lower concentrations [90–92]. Antibodies used for blocking, and matched isotype controls, were all low-endotoxin, azide-free (LEAF) and purchased from Biolegend: anti-NKG2D (clone 1D11, used at 50 μg/ml) with isotype (mouse IgG1, clone MOPC-21), anti-IL10R (clone 3F9, used at 20 μg/ml) with isotype (rat IgG2a, clone RTK2758), anti-IL10 (clone JES3-9D7, used at 20 μg/ml) with isotype (rat IgG1, clone RTK2071), anti-PD-1 (clone EH12.2H7, used at 10 μg/ml) with isotype (mouse IgG1, clone MOPC-21). Investigation of the recognition by CD8+ T cell clones of WT and ΔLMP2A LCLs established from the same donor was always performed in parallel and for at least one WT line and one ΔLMP2A line. Statistical analysis was performed with GraphPad Prism software.

The cytotoxic reactivity of CD8+ T cell clones against target cells was measured by calcein-release assay. Target cells (4×10^5^) were labeled with 1 μg/ml in 500 μl medium. After incubation for 1 hour at 37°C cells were washed 3 times with sterile PBS, and 5×10^3^ target cells/well were plated in a V-bottom 96-well plate (final volume 200 μl/well). For each target cell type, spontaneous release (no effector cells, 0% lysis) and maximal release (addition of 0.5% of triton-X 100, 100% lysis) was determined. Effector cells were co-incubated with target cells for 3 hours at a 2:1 ratio. Afterwards, 100 μl of supernatant were collected and transferred to a fresh flat-bottom 96-well plate and fluorescence intensity at 485/535 nm was measured in an Infinite F200 PRO fluorometer (Tecan). RPMI without phenol red was used to reduce background fluorescence.

### Quantitative RT-PCR

Total RNA was extracted from LCLs with the RNeasy Mini Kit, and cDNA synthesis was performed with the QuantiTect kit, both from Qiagen, Hilden, Germany. Quantitative PCR was performed on a LightCycler 480 (Roche, Basel, Switzerland) using the SYBR Green LC480 Mix. Primers were as follows: human IL-10 (forward: 5'-GCAGGTGAAGAATGCCTTTA-3', reverse: 5'-CCCTGATGTCTCAGTTTCGT-3'), BZLF1 unspliced (forward: 5'-GCACATCTGCTTCAACAGGA-3', reverse: 5'-CCAAACATAAATGCCCCATC–3'), EBNA1 (forward: 5'-CGCAAGGAATATCAGGGATG-3', reverse: 5'-TCTCTCCTAGGCCATTTCCA-3'), gp350 (forward: 5'- TTGTGAAATTTCGCCATCCT-3', reverse: 5'-CAAAACCCCGTGTACCTG-3'). Primers specific for BCRF1 (vIL-10), EBNA3A, LMP2AB, and GUSB were described before [42]. Specific mRNA expression was standardized to the housekeeping gene β-glucuronidase (GUSB) [91,93]. WT and ΔLMP2A LCLs simultaneously established from the same donor were always analyzed in parallel.

### Analysis of protein levels

Cells were incubated for 15 min on ice with lysis buffer (50 mM Tris/HCl pH 7.4, 150 mM NaCl, 1% NP40, 0.5% DOC, 0.1% SDS) together with protease inhibitor (completeMini, Roche). Protein concentration was determined with the Bio-Rad Protein Assay. Proteins were separated on an 8% SDS-PAGE gel and transferred to a nitrocellulose membrane by semi-dry blotting. Blots were probed with antibodies specific for LMP1 (1G6, provided by Elisabeth Kremmer, 1:25 dilution) [94] and GAPDH (1A7, 1:10 dilution). Blots were further probed with secondary antibodies conjugated to horseradish peroxidase, and immunoreactive proteins were detected by incubation with chemoluminescence substrate (0.1M Tris/HCl, pH 8.8, 200 mM p-Coumaric Acid in DMSO, 1.25 mM Luminol in DMSO) and exposure of CEA RP NEW films (Agfa HealthCare, Belgium).

## Supporting Information

S1 Fig Analysis of apoptosis and proliferation in WT and ΔLMP2A LCLs. (A) Gating strategy for the analysis of apoptotic cells. The triangular gate identifies early apoptotic cells, the rectangular gate late apoptotic cells. (B) Apoptosis and proliferation in WT and ΔLMP2A LCLs. For each of 4 different donors, two WT and two ΔLMP2A LCLs were investigated. Cells were stained with CellTrace Violet on day 0, and with annexin V and PI on day 4 of cultivation. The bottom left panel shows the mean fluorescent intensity (MFI) of all viable cells on day 4, the bottom right panel shows the ratio of MFI on day 4 divided by MFI on day 0. Statistical analyses were performed with the Mann-Whitney U test. One representative of two independent experiments is shown.
(TIFF)

S2 Fig Cytotoxic activity of HCMV-specific CD8+ T cell clones against LCLs with or without LMP2A. WT and ΔLMP2A LCLs were loaded with a 10^–8^ mol/L of the NLV peptide from the protein pp65 of HCMV and used in a cytotoxicity assay with NLV-specific CD8+ T cell clones. LCLs from 3 donors were used as targets for two NLV-specific CD8+ T cell clones at an effector:target ratio of 2:1. Statistical analysis was performed with the Wilcoxon test.
(TIFF)
