# Supplementary figures and images for: Latent Membrane Protein LMP2A Impairs Recognition of EBV-Infected Cells by CD8+ T Cells
Source: PLoS Pathog. 2015 Jun 11;11(6):e1004906. doi: 10.1371/journal.ppat.1004906 (PMC4465838; doi:10.1371/journal.ppat.1004906)

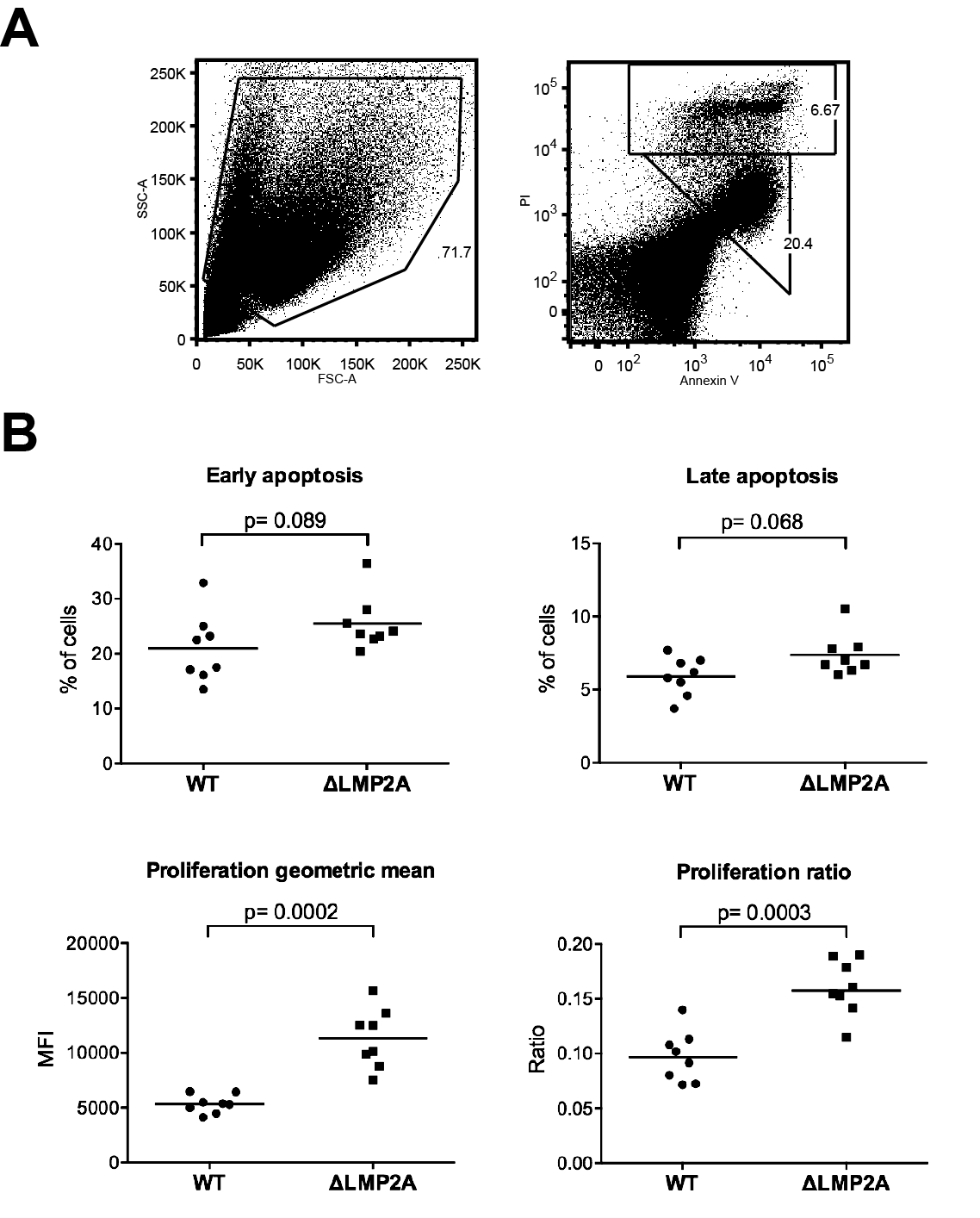

Supplement: S1 Fig — (A) Gating strategy for the analysis of apoptotic cells. The triangular gate identifies early apoptotic cells, the rectangular gate late apoptotic cells. (B) Apoptosis and proliferation in WT and ΔLMP2A LCLs. For each of 4 different donors, two WT and two ΔLMP2A LCLs were investigated. Cells were stained with CellTrace Violet on day 0, and with annexin V and PI on day 4 of cultivation. The bottom left panel shows the mean fluorescent intensity (MFI) of all viable cells on day 4, the bottom right panel shows the ratio of MFI on day 4 divided by MFI on day 0. Statistical analyses were performed with the Mann-Whitney U test. One representative of two independent experiments is shown. (TIFF) [file ppat.1004906.s001.tiff]

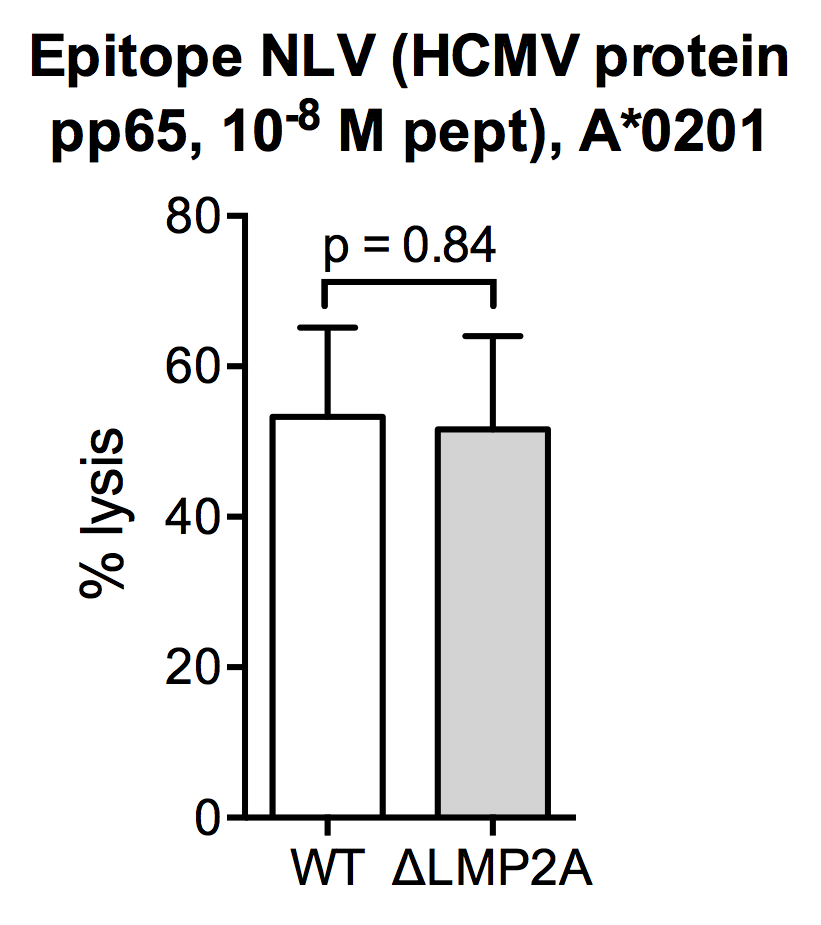

Supplement: S2 Fig — WT and ΔLMP2A LCLs were loaded with a 10–8 mol/L of the NLV peptide from the protein pp65 of HCMV and used in a cytotoxicity assay with NLV-specific CD8+ T cell clones. LCLs from 3 donors were used as targets for two NLV-specific CD8+ T cell clones at an effector:target ratio of 2:1. Statistical analysis was performed with the Wilcoxon test. (TIFF) [file ppat.1004906.s002.tiff]
